# Are Small Animal Practitioners Occupationally Exposed to Leptospirosis? Results of a Serological Survey

**DOI:** 10.3390/ijerph19031797

**Published:** 2022-02-04

**Authors:** Elisa Mazzotta, Laura Lucchese, Cristiano Salata, Tommaso Furlanello, Ermenegildo Baroni, Alessandro Zotti, Gabriele Venturi, Alice Fincato, Silvia Marchione, Katia Capello, Alda Natale

**Affiliations:** 1 Istituto Zooprofilattico Sperimentale delle Venezie, 35020 Legnaro, Italy; emazzotta@izsvenezie.it (E.M.); llucchese@izsvenezie.it (L.L.); afincato@izsvenezie.it (A.F.); smarchione@izsvenezie.it (S.M.); kcapello@izsvenezie.it (K.C.); 2 Department of Molecular Medicine, University of Padova, 35121 Padova, Italy; cristiano.salata@unipd.it; 3 Clinica Veterinaria San Marco, 35030 Veggiano, Italy; tf@sanmarcovet.it; 4 Clinica Veterinaria Baroni, 45100 Rovigo, Italy; gildo.baroni@clinicaveterinariabaroni.com; 5 Department of Animal Medicine, Production and Health, University of Padova, 35020 Legnaro, Italy; alessandro.zotti@unipd.it; 6 Freelance Small Animal Practitioner, 40126 Bologna, Italy; gabrinhood@yahoo.it

**Keywords:** Leptospira antibodies, human, people, microagglutination test (MAT), occupational exposure

## Abstract

Leptospirosis is a worldwide zoonosis frequently responsible for clinical disease in dogs and rarely reported in human people. The risk of human exposure to Leptospira has been investigated in a sample population working in the northeast of Italy, a geographical area with high endemicity of canine leptospirosis. Two-hundred twenty-one human serum samples were analyzed for Leptospira microagglutination test (MAT): 112 clinical freelance small animal practitioners (exposed subjects) and 109 people not occupationally exposed to Leptospira-infected animals (unexposed subjects) were voluntarily enrolled. Despite the previously reported serological detection of antibodies vs. Leptospira in people in different Italian regions, this study did not detect any reactivity in the investigated population. This study shows that veterinarians do not appear to be at a greater risk of leptospirosis than the reference population. This may be due to both veterinarian awareness of the Leptospira zoonotic risk and the efficiency of the preventive measures and management of patients. Moreover, it could be the result of the relatively low excretion of Leptospira in symptomatic dogs, which can be considered as an environmental sentinel for Leptospira presence rather than a vehicle of transmission.

## 1. Introduction

Leptospirosis is a neglected zoonosis, although one of the most widespread in the world. It is currently considered a re-emerging pathology linked to the interaction between people and various environmental factors and contact with animals (travel, outdoor recreational activities, sports, contact with reservoir animals, and occupational exposure). Globally, approximately one million Leptospira infections in humans, particularly in tropical and subtropical countries [1,2,3], are reported each year, with nearly 60,000 deaths [4], making leptospirosis a significant threat to global public health [5]. In Europe, the prevalence of the disease is potentially underestimated due to the scarcity of official reports [6]. In particular, the real prevalence of Leptospira human infection in Italy seems to be frequently underestimated, and a few clinical cases were officially reported in people over the last decade [7,8]. Previous studies aimed to assess the seroprevalence of Leptospira among residents in different Italian regions, and they reported a broad variability of results [9,10,11]. In a study conducted from 1995 to 2001 in Northern and Central Italy, a 5.6% positivity for antibodies against Leptospira in human sera was reported in people undergoing diagnostic investigations in which the differential diagnosis for leptospirosis was included [11]. In 1994, a serological survey reported a total seroprevalence of 12% in people in different Italian regions [10]. More recently in 2009, no serological reactivity against Leptospira was reported both for farm workers and control group in a study conducted in Bari (Apulia, Southern Italy) [9]. The epidemiological situation of Leptospirosis in the northeast of Italy is worthy of attention, as it is a widespread zoonosis in the synanthropic fauna, particularly in the rat and hedgehogs, with frequent reports of clinical cases in dogs [12] but rarely in humans. In August 2002, after an exceptional flood in Vicenza (North-East of Italy) a local seroepidemiological survey reported a 6.8% seroconversion rate in the population exposed to the inundation [13]. Furthermore, in 2004, a study reported a seroprevalence of 13.74% in farmers resident in different municipalities around the Brenta river (Pedemontano Brenta consortium area, northeast of Italy) [14]. Recently, few cases of Leptospira infections in people were reported in Venice and in Sicily (Palermo) [7,15]. These previous studies suggested that the higher occupational risk was related to environmental factors (water, soil, etc.) rather than to the direct contact with infected animals. The present study aims to evaluate the risk of exposure to Leptospira infection in a sample population of professionally exposed people and not occupationally exposed subjects. Our findings suggest that Leptospira infection seems to have a very low prevalence in people in the studied area, even considering professional that are likely exposed to the infection. 

## 2. Materials and Methods

### 2.1. Sample Population

The study was performed in years 2019–2020 on people occupied in the northeast of Italy. The participants were 221 persons, 59 male, and 162 female aged between 23 to 65 years old. The population was divided into 2 groups:

- N. 112 veterinarian small animal practitioners, as subjects exposed to professional risk due the contact with biological material potentially infected (blood, urine, …) and clinically ill dogs in a geographic area with high endemicity for canine leptospirosis (exposed population); no other health professionals or collaborators (e.g., zootechnical practitioners, shelter workers, kennel volunteers) were included in this category;
- N. 109 people living in the same geographic area, not occupationally exposed to Leptospira infected animals (control population).

The sample size of the two groups was preventively established considering mainly different factors: the feasibility of the study, the non-invasiveness of the medical procedure, and the limited literature reports about leptospiral infection in human population. Nevertheless, the identified sample size allowed achieving a statistical power at least equal to 95% in the case of prevalence for the exposed group greater than 13% that is the estimated prevalence reported for an environmentally exposed group in a study conducted in the same area in 2004 [14]. Each volunteer was familiarized about the study’s characteristics, and the informed consent forms for the authorization to use personal data as well as the privacy notices were signed and collected. In addition, an epidemiological questionnaire for the assessment of the exposure to potential leptospirosis risk factors was submitted to all participants (Appendix A). The form included personal data (municipality of residence, age, and sex), information regarding the inhabited site (urban or rural), work activity (office, laboratory, and veterinarian clinical activity), and frequency of exposure to domestic, synanthropic, or wild animals (livestock, pets, and local fauna). In particular, dog owners were asked for their dogs’ epidemiological information: usual location and life site (indoor or outdoor, country or city), access to river/water courses, vaccinations against leptospirosis, contacts with other pets, domestic or wild animals, eventually medical history of previous Leptospira infection.

The documents provided to the participants were approved by the local Ethics Committee (prot. N. 00065353).

The blood samples were collected by healthcare operators during predisposed sessions: for each participant, 8 mL of blood was collected in sterile tubes (BD Vacutainer^®^, Becton Dickinson, Oxford, UK) and centrifuged within 60 min in order to harvest serum that was kept refrigerated until analysis.

### 2.2. Serological Analysis

Serum analyses were performed by using the MAT method, applying the diagnostic protocol for 11 antigens panel routinely used for the diagnostic in dogs. MAT was performed following the OIE Manual of Diagnostic Tests and Vaccines for Terrestrial Animals 2021 (Chapter 3.1.12) [16], with a minimum dilution of serum 1:100. The antigen panel included 8 serogroups and 11 serovars (sv) distributed by the Italian Reference Center for Animal Leptospirosis as antigens in the routine diagnostic microagglutination test, representative of those known to exist in the Italian area: Icterohaemorrhagiae sv Icterohaemorrhagiae (strain Bianchi); Icterohaemorrhagiae sv Copenagheni (strain Wijnberg n. 1); Australis sv Bratislava (strain Riccio 2 n. 47); Canicola sv Canicola (strain Alarik n. 2); Sejroe sv Hardjo (strain Hardjoprajitno n. 224); Sejroe sv Sejroe (strain M84); Sejroe sv Saxkoebing (strain Mus24); Tarassovi sv Tarassovi (strain Mitis Johnson n. 6); Grippothyphosa sv Grippotyphosa (strain Moska V n. 54); Pomona sv Pomona (strain Pomona n. 222); and Ballum sv Ballum (strain Mus 127 n. 217).

### 2.3. Statistical Analysis

The results of the serological tests were subjected to statistical analysis in order to compare the levels of seroprevalence between the two groups and to evaluate the association with the identified possible risk factors. The *t*-test was used to evaluate the mean age in the two groups after having evaluated the homogeneity of variances. The chi-squared test or Fisher’s exact test were applied to analyze the identified factors. Given the results of the bivariate analyses and the observed frequencies, no multivariate analysis was performed. All statistical analyses were performed using the software STATA v 12.1.3.

## 3. Results

No serological reactivity to Leptospira serovars was detected by the MAT antigenic panel, both in exposed and unexposed groups. Nevertheless, the sample size and results, particularly for the exposed group, allow considering a 99% confidence level, which is the population free from disease at a minimum prevalence level of 5%. 

Table 1 showed the results of the analyses of data collected through the questionnaire in comparison with the groups (Table 1). Significant values (*p* < 0.01) were detected for the average age between groups, with lower mean values in the unexposed group. The exposed group highlighted a significant high frequency about habitual animal’s contacts (pets and wild animals). Animal ownership was significantly more frequent (*p* < 0.001) in the exposed group: 59.8% of subjects declared to have at least one dog versus 37.6% in the unexposed group. In addition, 64.3% of the exposed subjects declared to own other domestic animals, such as cats, horses, cows, rabbits, goats, and sheep, versus 37.6% in the unexposed group. No statistical differences were observed between the two groups regarding the Leptospira risk of exposure of their dogs (Table 2).

## 4. Discussion

Leptospirosis is an infectious disease significantly reported in veterinary practice and worldwide. To provide an idea of its scope, in one veterinary practice where a part of the sampled professionals had their working site, there was an average caseload of 60 cases of canine leptospirosis/year (T. Furlanello, unpublished information). Dealing with clinical cases, many clinic’ staff members came into contact with the hospitalized subjects.

The present study did not report any serological reactivity against Leptospira in people occupationally exposed (veterinarians) and unexposed (control population) in the northeast of Italy. This result can be due to good veterinary practices such as the use of adequate personal protections and specific management of animals with a clinical suspect of Leptospira infection. In addition, despite animal ownership being significantly more frequent in the exposed group, no statistical differences were observed between the two groups regarding the Leptospira risk of exposure in own dogs. Reasonably, the risk of Leptospira infection in unexposed group is lower because of the expected good health state of their pets, rather than the veterinarian’s occupational management of sick and infected animals. Comparable results have been reported in a recent study conducted on veterinary staff and dogs’ owners exposed to dogs with acute leptospirosis, which described no seroreactivity to Leptospira serovars [17]. According to previous literature [9,11,14], this study suggests that domestic animals, particularly dogs, may play a role as a sentinel of infection rather than a source of contagion for humans. In particular, the low shedding and survival of leptospires in infected dog’s urine, reported around 6 to 8% [11,18], could represent a low risk to be infected through the dog’s urine for exposed people [19]. Finally, according to previous studies [9,14], the serological prevalence of Leptospira antibodies in occupationally exposed people seems to be more likely associated to environmental factors (water, soil, etc.) than to direct contact with pet animals. The recreational activity, travels, exposure to environmental Leptospira reservoirs (wild and synanthropic animals), and access to freshwater sources are not reported as significant in our study. The exposed group reported a significantly higher percentage of dog’s ownership (59.8%), although the dog’s contact associated risk factors (Table 2) are not significant. Moreover, in the exposed group, a high percentage of owned dogs were vaccinated against Leptospira (83.6%). Until now, vaccination against Leptospira is recommended in order to prevent the severe clinical diseases in dogs, more commonly caused by serovar Icterohaemorrhagiae in the northeast of Italy [12]. Furthermore, the most recent studies reported that vaccination of dogs with the new anti-Leptospira vaccine induces protective immunity against systemic disease, renal infection, and clinical signs [20], even if the available commercial vaccine formulations are not able to protect the dogs from Leptospira infection caused by emergent serogroups (Pomona, Sejroe) [21,22,23,24]. The widespread emergence of Leptospira strains requires the employment of specific diagnostic methods (MAT, molecular analysis, and genotyping) in order to better understand geographical distribution and to develop appropriate vaccine formulations. In this study, no data of concern have emerged, although it is pivotal to consider the risk of persistent leptospiral shedding in asymptomatic/paucisymptomatic dogs. Veterinarians may be less aware of the risk of Leptospira infection consequent to direct contact with these subjects [25,26].

## 5. Conclusions

Leptospirosis is reported as a worldwide spread zoonosis, despite it remaining a neglected disease. In Italy, Leptospira infection is widely spread in synanthropic and wild animals (rodents) and causes relatively frequent and serious, sometimes fatal, clinical diseases in dogs. In addition, there are sporadic officially reported cases in people, which would make the incidence of the disease underestimated. The preset study evaluates the occupational risk of exposure to Leptospira in small animal practitioners working the North East of Italy by conducting a serological survey: 112 professionally exposed subjects and 109 people not occupationally exposed to Leptospira infected animals were serological evaluated for antibodies vs. Leptospira. No serological positivity was reported in the investigated population, both small animal practitioners and not exposed population. This study shows that veterinarians do not appear to be at greater risk of leptospirosis than the reference population. Consequently, the present study suggests that Leptospira infection seems to have a very low prevalence in people in the studied area, even considering that they are likely exposed to the infection. According to previous literature, the infection seems to be more frequent in environmentally exposed people than in subjects occupationally exposed to ill dogs. Therefore, the dog seems to play a role as environmental sentinels rather than a source of infection for people. The changing climatic situation, the increase in average temperatures in cold season, and the increasingly frequent weather phenomena such as prolonged rainfall and floods raise concerns about the possible increased risk of diffusion of this zoonosis. In a One Health vision, further studies are necessary to better describe the risk for the human population, ideally reporting a zoonotic risk map based on case reports in dogs and people and surveillance in synanthropic and wild animal species.

## Figures and Tables

**Table 1:** Evaluation of risk factors between occupationally exposed and unexposed groups. Ages are reported in years mean values ± standard deviation. Each factor was expressed by the sample size (N) and the percentage (%). *p* values <0.05 were considered significant. *p* values >0.05 were considered not significant (NS).

Risk Factor	Exposed N (%)	Unexposed N (%)	*p*-Value
Age (years)	44.4 ± 9.8	39.6 ± 9.7	<0.001
Sex			
F	82 (73.2%)	80 (73.4%)	NS
M	30 (26.8%)	29 (26.6%)	
Residence			
Urban/town	65 (58.0%)	55 (50.1%)	NS
Village	26 (23.2%)	32 (29.6%)	
Rural	16 (14.3%)	20 (18.5%)	
**Animal contacts**			
Pets			
Frequent	106 (94.6%)	62 (56.9%)	<0.001
Occasional	6 (5.4%)	18 (16.5%)	
No	0	29 (26.6%)	
Farm animals			
Frequent	4 (3.6%)	2 (1.8%)	NS
Occasional	19 (16.9%)	12 (11.0%)	
No	89 (79.5%)	95 (87.2%)	
Wild animals			
Frequent	2 (1.8%)	1 (0.9%)	0.025
Occasional	18 (16.1%)	7 (6.4%)	
No	92 (82.1%)	101 (92.7%)	
Dog ownership			
Yes	67 (59.8%)	41 (37.6%)	0.001
No	45 (40.2%)	68 (62.4%)	
Other animals ownership			
Yes	72 (64.3%)	40 (37.6%)	<0.001
No	41 (35.7%)	68 (62.4%)	

**Table 2:** Evaluation of Leptospira risk of exposure in owned dogs between professionally exposed and unexposed dog’s owners. Each factor was expressed by the sample size (N) and the percentage (%). *p* values <0.05 were considered significant. *p* values >0.05 were considered not significant (NS).

Owned Dog Risk Factors	Exposed N (%)	Unexposed N (%)	*p*-Value
Usual inhabit			
Indoor	54 (80.6%)	31 (75.6%)	NS
Indoor/outdoor	8 (11.9%)	4 (9.7%)	
Outdoor	5 (7.5%)	6 (14.6%)	
Wild/domestic animals contacts			
Yes	56 (83.6%)	34 (82.9%)	NS
No	11 (16.4%)	7 (17.1%)	
Access to environmental freshwater sources			
Frequent	25 (37.3%)	10 (24.4%)	NS
Occasional	22 (32.8%)	16 (39.0%)	
No	20 (29.8%)	15 (36.6%)	
Vaccination against *Leptospira* spp.			
Yes	56 (83.6%)	29 (70.7%)	NS
No	11 (16.4%)	12 (29.3%)	

## Data Availability

All the data are available in this paper.

## Appendix A

Epidemiological Questionnaire
